# Supplementary material for: The receptor PTPRU is a redox sensitive pseudophosphatase
Source: Nat Commun. 2020 Jun 26;11:3219. doi: 10.1038/s41467-020-17076-w (PMC7320164; doi:10.1038/s41467-020-17076-w)
Supplement: Supplementary file 1 — Supplementary Information [file 41467_2020_17076_MOESM1_ESM.pdf]

Supplementary Information

The receptor PTPRU is a redox sensitive pseudophosphatase

Iain M. Hay, Gareth W. Fearnley, Pablo Rios, Maja Köhn, Hayley J. Sharpe and Janet E. Deane

Supplement contains 10 supplementary figures, 3 supplementary tables and supplementary references.

Family	PTP		pTyr-loop		WPD-loop		PTP-loop
R1	*PTPRC*	*677-686*	NQNKNRYVDI	*816-824*	FTSWPDHGV	*850-861*	VVHCSAGVGRTG
R2B	*PTPRM*	*923-932*	NRMKNRYGNI	*1058-1066*	FTGWPDHGV	*1092-1103*	VVHCSAGAGRTG
	PTPRK	*911-920*	NRAKNRYGNI	*1052-1060*	FTGWPDHGV	*1086-1097*	VVHCSAGAGRTG
	PTPRT	*912-921*	NRNKNRYGNI	*1047-1055*	FTSWPDHGV	*1081-1092*	VVHCSAGAGRTG
	PTPRU	*913-922*	KVKGSRQEPM	*1048-1056*	FTAWPEHGV	*1082-1093*	VIHCSAGTGRTG
R2A	*PTPRF*	*1375-1384*	NKPKNRYANV	*1511-1519*	FMAWPDHGV	*1545-1556*	VVHCSAGVGRTG
	PTPRS	*1416-1425*	NKPKNRYANV	*1552-1560*	FTAWPDHGV	*1586-1597*	VVHCSAGVGRTG
	PTPRD	*1380-1389*	NKPKNRYANV	*1516-1524*	FTAWPDHGV	*1550-1561*	VVHCSAGVGRTG
R4	*PTPRA*	*265-274*	NKEKNRYVNI	*405-413*	FTSWPDFGV	*439-450*	VVHCSAGVGRTG
	PTPRE	*159-168*	NREKNRYPNI	*298-306*	FTSWPDFGV	*332-343*	VVHCSAGVGRTG
R5	*PTPRG*	*874-883*	NKHKNRYINI	*1023-1031*	YTQWPDMGV	*1057-1068*	LVHCSAGVGRTG
	PTPRZ	*1750-1759*	NKHKNRYINI	*1896-1904*	YTQWPDMGV	*1930-1941*	VVHCSAGVGRTG
R3	*PTPRB*	*1727-1736*	NRGKNRYNNI	*1865-1873*	YTVWPDHGV	*1901-1912*	VVHCSAGVGRTG
	PTPRJ	*1065-1074*	NRGKNRYNNV	*1200-1208*	FTSWPDHGV	*1236-1247*	LVHCSAGVGRTG
	PTPRH	*844-853*	NNAKNRYRNV	*981-989*	YQAWPDHGV	*1017-1028*	IVHCSAGVGRTG
	PTPRO	*962-971*	NRCKNRYTNI	*1097-1105*	YTAWPDHGV	*1133-1144*	IIHCSAGVGRTG
	PTPRQ	*2060-2069*	NRAKNRFPNI	*2196-2204*	FTAWPEHGV	*2230-2241*	IVHCSAGVGRTG
R7	*PTPRR*	*415-424*	HGTKNRYKTI	*549-557*	YTSWPDHKT	*585-596*	VVHCSAGIGRTG
	PTN5	*322-331*	LVRKNRYKTI	*456-464*	FTSWPDQKT	*493-504*	IVHCSAGIGRTG
R8	*PTPRN*	*734-743*	NIKKNRHPDF	*872-880*	FLSWPAEGT	*906-917*	IVHCSDGAGRTG
	PTPRN2	*770-779*	NVPKNRSLAV	*908-916*	FLSWYDRGV	*942-953*	IVHCSDGAGRSG
NT1	*PTN1*	*40-49*	NKNRNRYRDV	*176-184*	YTTWPDFGV	*212-223*	VVHCSAGIGRSG
	PTN2	*42-51*	NRNRNRYRDV	*177-185*	YTTWPDFGV	*213-224*	VIHCSAGIGRSG
NT2	*PTN6*	*270-279*	NKGKNRYKNI	*414-422*	YLSWPDHGV	*450-461*	IVHCSAGIGRTG
	PTN11	*273-282*	NKNKNRYKNI	*424-432*	FRTWPDHGV	*460-471*	VVHCSAGIGRTG
NT3	*PTN9*	*327-336*	NLEKNRYGDV	*465-473*	FLSWPDYGV	*512-523*	VVHCSAGIGRTG
NT4	*PTN18*	*56-65*	NVRKNRYKDV	*192-200*	YMSWPDRGV	*226-237*	CVHCSAGCGRTG
	PTN12	*58-67*	NVKKNRYKDI	*194-202*	YVNWPDHDV	*228-239*	CIHCSAGCGRTG
	PTN22	*54-63*	NIKKNRYKDI	*190-198*	YKNWPDHDV	*224-235*	CIHCSAGCGRTG
NT5	*PTN3*	*670-679*	NLDKNRYKDV	*806-814*	YVAWPDHGV	*839-850*	LVHCSAGIGRTG
	PTN4	*679-688*	NISKNRYRDI	*815-823*	YIAWPDHGV	*849-860*	VVHCSAGIGRTG
NT6	*PTN21*	*921-930*	NAERNRFQDV	*1062-1070*	YTDWPEHGC	*1105-1116*	LVHCSAGVGRTG
	PTN14	*933-942*	NAERSRIREV	*1074-1082*	YTDWPDHGC	*1118-1129*	VVHCSAGVGRTG
NT7	*PTN13*	*2237-2246*	NRRKNRYKNI	*2373-2381*	FTAWPDHDT	*2405-2416*	ITHCSAGIGRSG
NT8	*PTN23*	*1217-1226*	YSLKNRHQDV	*1352-1360*	FPTWPELGL	*1389-1400*	IVHCSSGVGRTG
NT9	*PTN20*	*183-192*	NREKNRYRDI	*318-326*	FTKWPDHGT	*350-361*	VVHCSAGIGRTG
	PTN7	*119-128*	HASKDRYKTI	*252-260*	FSAWPDHQT	*288-299*	VVHCSAGIGRTG

Supplementary Fig. 1. Multiple sequence alignment of the pTyr recognition loop, WPD loop and PTP loop of the 37 classical PTPs, coloured by percentage identity (blue). Key variable residues in PTPRU are highlighted in red.

PTPRU D1 domain:

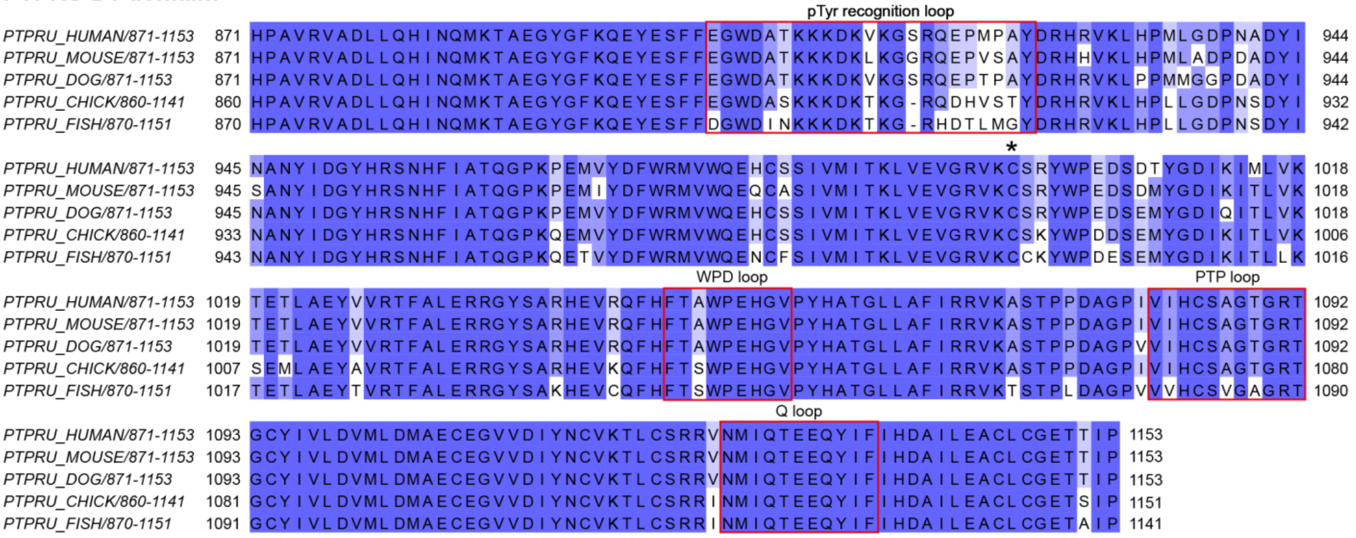

Supplementary Fig. 2. Multiple sequence alignment of PTPRU-D1 sequences from human (*Homo sapiens*), mouse (*Mus musculus*), dog (*Canis familiaris*), chicken *(Gallus gallus*) and zebrafish (*Danio rerio*). Sequences are coloured by percentage identity (blue). Key PTP motifs are labelled and boxed in red. The “backdoor” cysteine (C998) which upon oxidation forms an intramolecular disulphide with the catalytic C1085 of the PTP loop is highlighted (*).

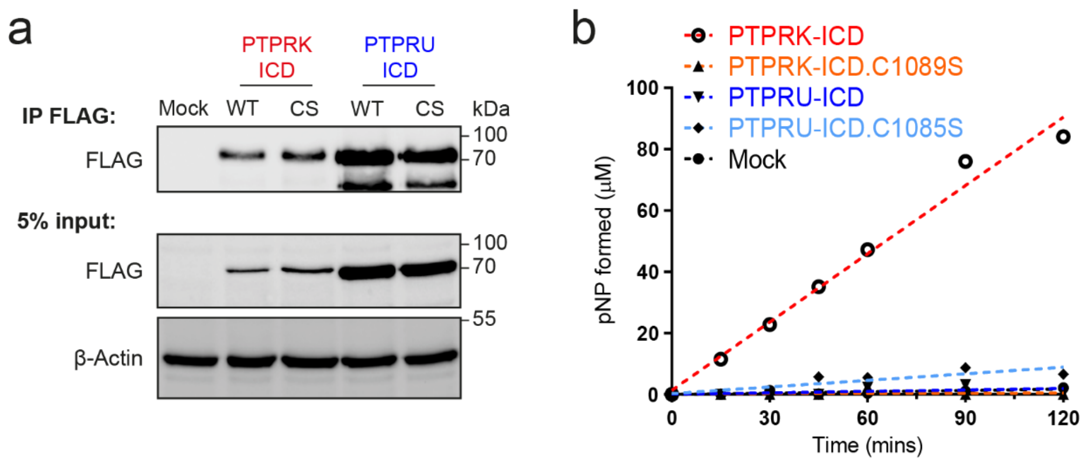

Supplementary Fig. 3. Replicate of immunoprecipitation (IP) and pNPP assays of FLAG-tagged PTP intracellular domains (ICD). a Immunoblot analysis of FLAG IPs from HEK-293T cells transiently transfected with PTPRK and PTPRU WT and CS inactivating mutant ICDs. **b** Time course of pNPP dephosphorylation by FLAG IPs from (**a**).

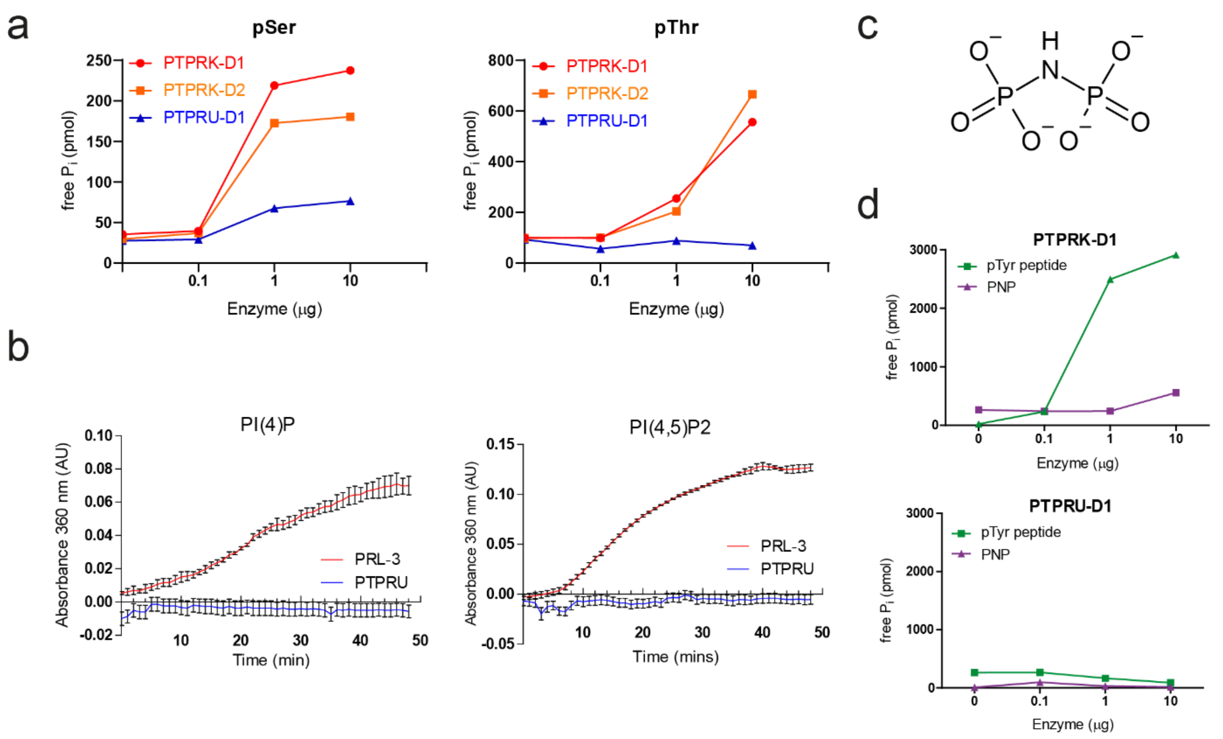

Supplementary Fig. 4. The PTPRU-D1 domain shows no activity against diverse substrates. a Activity of PTPRU-D1, PTPRK-D1 and PTPRK-D2 domains incubated with 100 μM of either pSer or pThr amino acids, measured using BIOMOL Green reagent. Note: the activity levels detected here are very low compared to that of a validated enzyme-substrate reaction (compare to Y axis of panel **d**). The equivalent activity of the PTPRK D1 and D2 domains suggests this level of activity is non-specific. **b** Phosphatidylinositol (PI) phosphatase activity assay. Phosphatidylinositol 4-phosphate [PI(4)P] and phosphatidyl 4,5-bisphosphate [PI(4,5)P2] substrates were incubated with either 3 μM PTPRU-D1 or 6 μM PRL-3 (positive control for PI phosphatase activity)[1] and product formation monitored by measurement of absorbance at 360 nm. **c** Chemical structure of the phosphoramidate-linked substrate imidodiphosphate (PNP). **d** Activity of PTPRU and PTPRK D1 domains incubated with 100 μM of either pTyr peptide or PNP, measured using BIOMOL Green reagent.

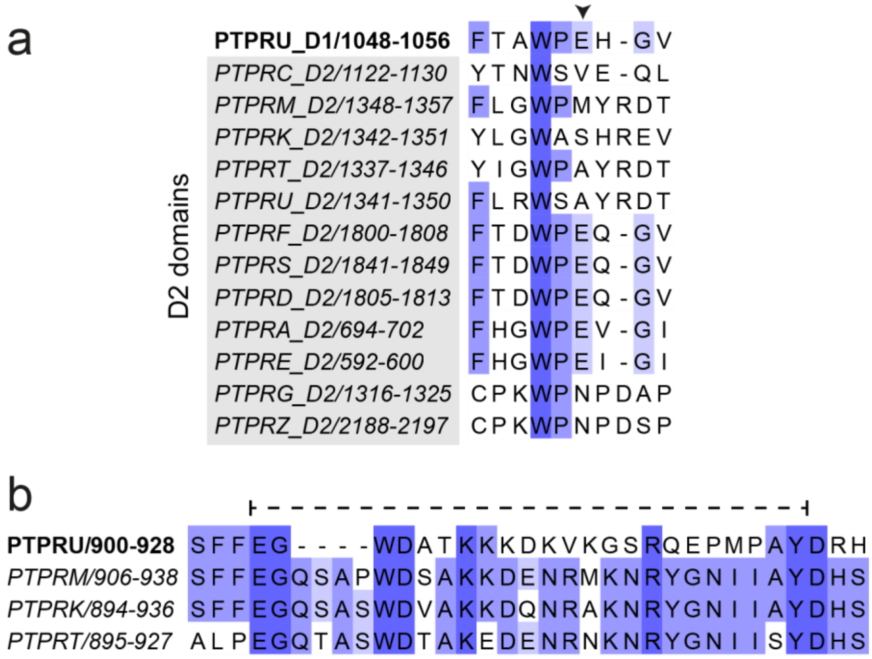

Supplementary Fig. 5. Sequence alignments of PTPRU catalytic motifs with relevant PTPs. **a** Multiple sequence alignment of the PTPRU-D1 WPD loop with those of the receptor PTP D2 domains, coloured by percentage identity (blue). The non-canonical glutamate of the PTPRU-D1 WPD-loop is marked by an arrowhead. **b** Multiple sequence alignment of the R2B family pTyr recognition loops coloured by percentage identity (blue). Residues 904-925 of PTPRU, which are disordered in the structure, are highlighted by a dashed line.

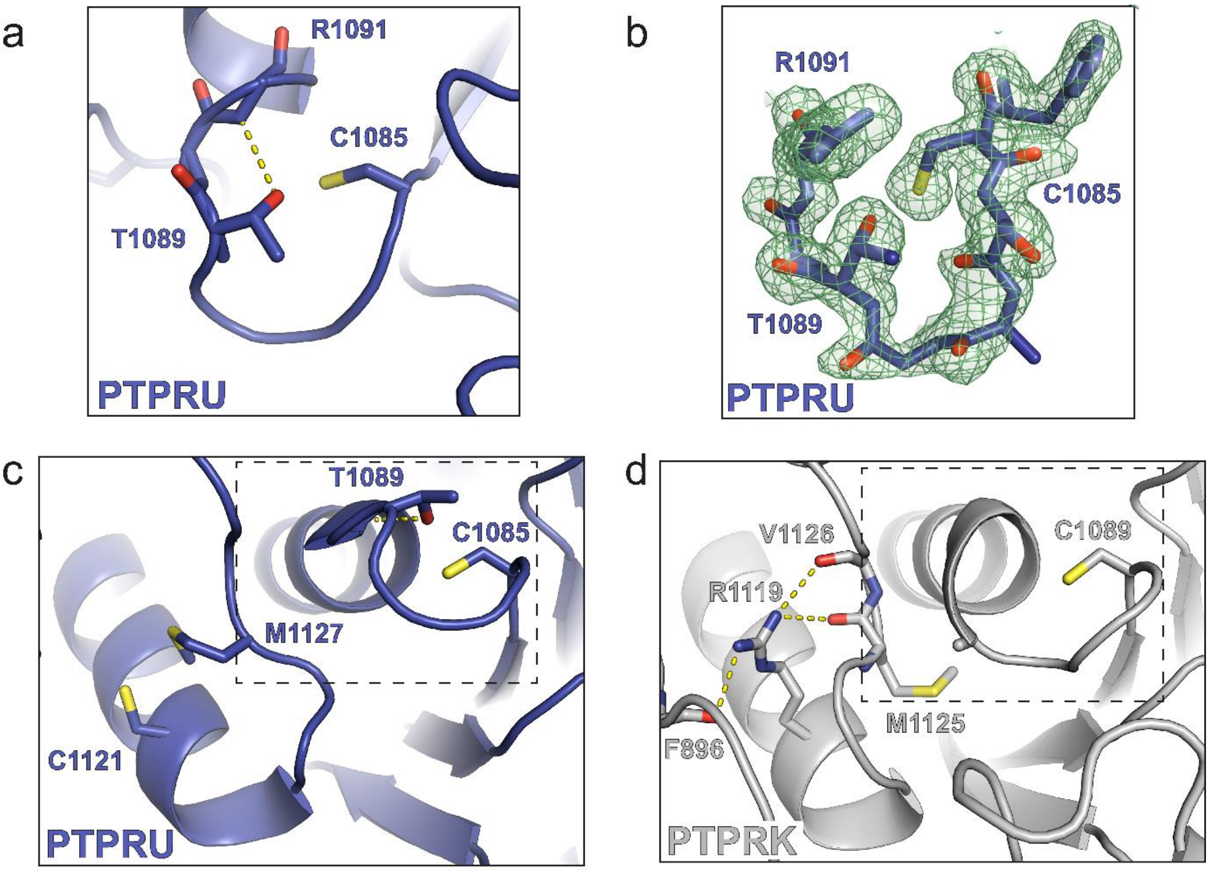

Supplementary Fig. 6. Conformational changes of the PTP and adjacent loops. a T1089 within the PTP loop is stabilised in a novel conformation via hydrogen bonding to the backbone of R1091, capping the end of the α-helix. Hydrogen bonds are illustrated by a dotted yellow line. For clarity only specific mainchain and sidechain atoms are shown to help illustrate relevant bonds and interactions. **b** Electron density ($2F_o$-F_c contoured at 0.8 e^- /$Å^3$, green) for the novel PTPRU-D1 PTP loop (H1084-R1091, blue sticks) conformation shown in (**a**). **c** In PTPRU, the loop C1121-M1127 adjacent to the PTP loop adopts a conformation that differs from classical PTPs due to re-orientation of M1127. This loop is not well ordered and was challenging to build in a single conformation suggesting it may adopt multiple conformations. The dotted inset box identifies the equivalent region as illustrated in Fig. 2d in the main text. **d** In PTPRK, the equivalent loop R1119-M1125 is stabilised via hydrogen bonding between the R1119 sidechain and backbone carboxyl groups of M1125 and V1126. As for panel (**c**), the dotted inset box identifies the equivalent region as illustrated in Fig. 2d.

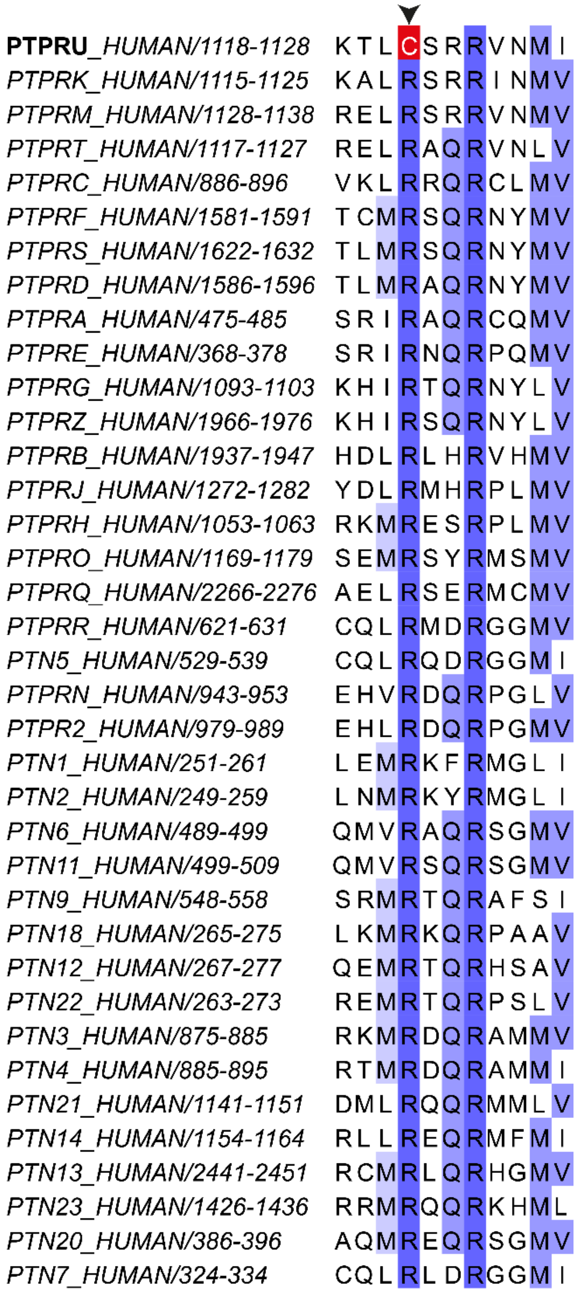

Supplementary Fig. 7. Multiple sequence alignment of PTPRU-D1 loop K1118-I1128 across the 37 classical PTPs, coloured by percentage identity (blue). C1121 (black arrowhead) in this loop is uniquely a cysteine in PTPRU.

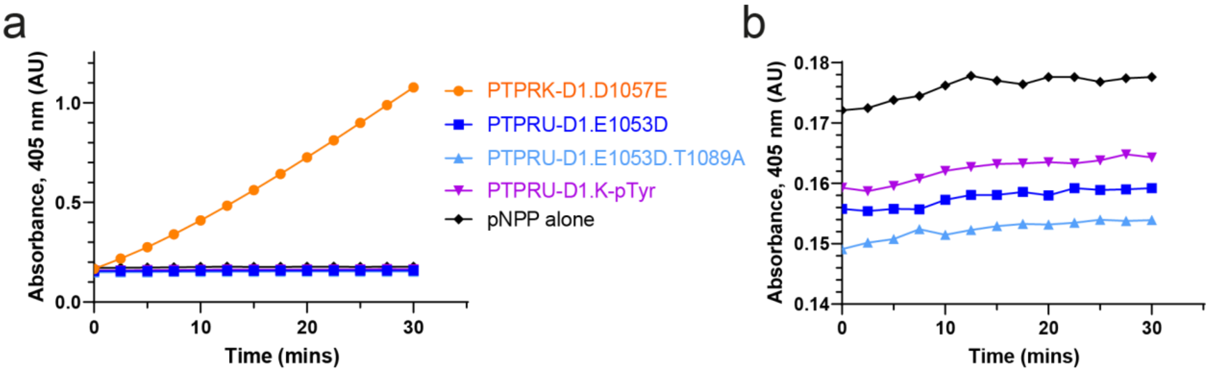

Supplementary Fig. 8. pNPP activity assays of PTPRU-D1 mutants. a Time course of pNPP dephosphorylation, monitored by absorbance at 405 nm, using 10 µM of PTPRK-D1.D1057E, PTPRU-D1.E1053D, PTPRU-D1.E1053D.T1089A and PTPRU-D1.K-pTyr recombinant proteins. **b** pNPP dephosphorylation data of inactive PTPRU-D1 mutants from (**a**). Background absorbance for all proteins does not exceed that observed for pNPP substrate incubated with assay buffer alone.

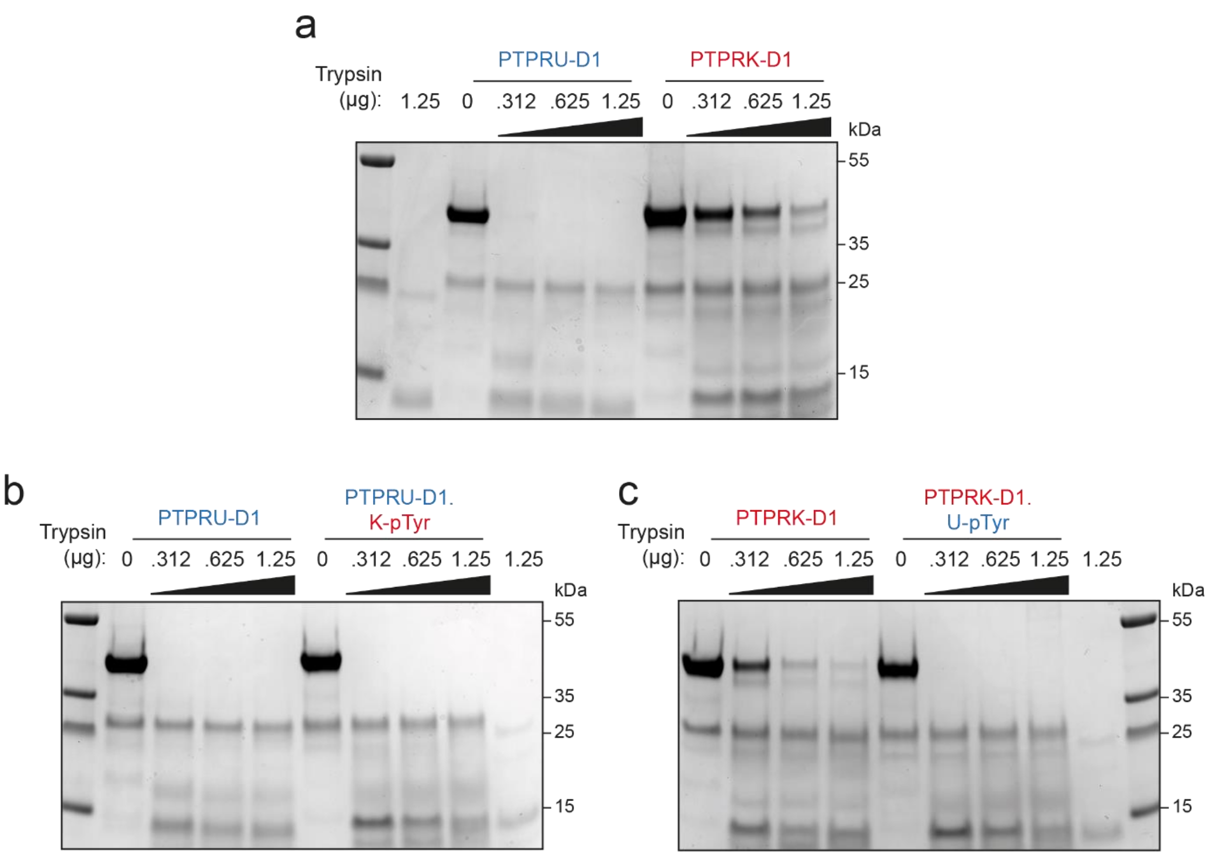

Supplementary Fig. 9. Trypsin limited proteolysis of the D1 domain pTyr recognition loop chimeras. Limited proteolysis of **a** PTPRU-D1 and PTPRK-D1, **b** PTPRU-D1 and PTPRU-D1.K-pTyr , **c** PTPRK-D1 and PTPRK-D1.U-pTyr with trypsin followed by SDS-PAGE and Coomassie staining.

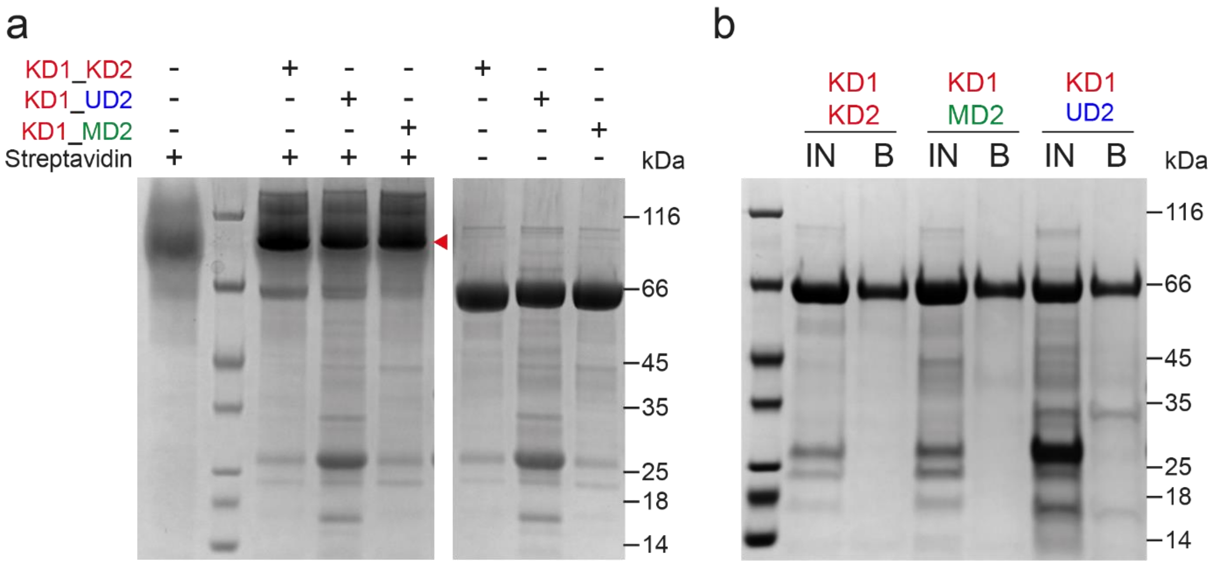

Supplementary Fig. 10. *In vivo* biotinylation of chimeric tandem PTP domains. **a** *In vivo* biotinylated chimeric tandem PTP domains incubated with or without streptavidin, resolved by SDS-PAGE and visualized by Coomassie staining. Mobility shift upon streptavidin binding to biotinylated protein is indicated by arrowhead. **b** *In vivo* biotinylated chimeric tandem PTP domains bound to streptavidin magnetic beads. Input protein (IN) and protein eluted from washed beads (B) was resolved by SDS-PAGE and visualized by Coomassie staining.

Supplementary Table 1. Percentage sequence identity matrix of R2B family D1 domains vs R2B family D1 (green) and D2 (yellow) domains. Generated by multiple sequence alignment using Clustal Omega[2].

	PTPRU D1	PTPRK D1	PTPRM D1	PTPRT D1	PTPRU D2	PTPRK D2	PTPRM D2	PTPRT D2
PTPRU D1	**100**	**72.44**	**69.61**	**64.66**	**27.34**	**29.2**	**28.15**	**26.64**
PTPRK D1	72.44	100	79.44	76.31	28.37	31.29	31.02	28.78
PTPRM D1	69.61	79.44	100	80.14	27.66	30.58	30.29	27.34
PTPRT D1	64.66	76.31	80.14	100	29.79	30.58	30.29	29.14

Supplementary Table 2. PTP domain structures used in structural alignments (Fig 2b and 2d).

PTP	PDB ID	RMSD (Å)*
PTPN5	2BIJ	1.0 over 1010 atoms
PTPN6	4HJP	1.0 over 1082 atoms
PTPN11	3B7O	1.0 over 1121 atoms
PTPN9	2PA5	0.9 over 1208 atoms
PTPN18	2OC3	1.2 over 1029 atoms
PTPN22	2P6X	1.2 over 1085 atoms
PTPN3	2B49	1.0 over 1139 atoms
PTPN4	2I75	1.1 over 1121 atoms
PTPN14	2BZL	1.1 over 1102 atoms
PTPN13	1WCH	1.0 over 1038 atoms
PTPRC	1YGU	1.1 over 1255 atoms
PTPRF	1LAR	0.8 over 1233 atoms
PTPRS	2FH7	0.9 over 1446 atoms
PTPRM	1RPM	0.8 over 1505 atoms
PTPRK	2C7S	0.8 over 1582 atoms
PTPRT	2OOQ	0.9 over 1500 atoms
PTPRB	2AHS	1.0 over 1337 atoms
PTPRJ	2NZ6	1.0 over 1216 atoms
PTPRO	2GJT	1.1 over 1332 atoms
PTPRA	1YFO	0.9 over 1334 atoms
PTPRE	2JJD	0.9 over 1204 atoms
PTPRG	2H4V	1.0 over 1416 atoms
PTPRR	2A8B	1.2 over 1170 atoms
PTPN7	2A3K	1.0 over 1095 atoms
PTPRN	2I1Y	1.1 over 1142 atoms
PTPRN2	2QEP	0.9 over 1062 atoms
PTPN1	2NT7	1.2 over 1116 atoms

*Calculated using extra_fit function within Pymol using default settings (5 cycles, cut-off = 2.0 Å) with PTPRU-D1 (reduced) as the target molecule.

Supplementary Table 3. Primer and oligonucleotide sequences used in this study.

Construct	Oligo (5'-3')*
PTPRU-D1 Fw	TGGC[GGTACC]CACCCTGCGGTG
PTPRU-D1 Rv	TGGC[ACTAGT]CTAAGGGATGGTGGTCTCCCCAC
PTPRU-ICD Fw	TGGC[TTCGAA]CGCAAAGGGAAGCCGGTGAAC
PTPRU-ICD Rv	TGGC[GGTACC]CTATCTTGACTCCAGCCCCTCCAAGTA
PTPRU.E1053D Fw	GCCAGATCATGGCGTCCCCTAC
PTPRU.E1053D Rv	CCATGATCTGGCCACGCTGTGAAG
PTPRU.C1085S Fw	CCACAGCAGCGCGGGC
PTPRU.C1085S Rv	CTGCTGTGGATGACAATGGGCC
PTPRU.T1089A Fw	GGGCGCCGGCCGCACAGGTTGCTATAT
PTPRU.T1089A Rv	GCCGGCGCCCGCGCTGCAGTGGATGACAAT
PTPRU-D1.ΔpTyr-loop Fw	GATCGGCACCGAGTGAAACTGC
PTPRU-D1.ΔpTyr-loop Rv	TTCAAAGAAGCTCTCATACTCCTGCTTGAAG
PTPRU-D1.pTyr loop oligo Fw	CTTTTTTGAAGGCTGGGACGCCACAAAGAAGAAAGACAAGGTCAAGGGCAGC CGGCAGGAGCCAATGCCTGCCTATGATCACT
PTPRU-D1.pTyr loop oligo Rv	AGTGATCATAGGCAGGCATTGGCTCCTGCCGGCTGCCCTTGACCTTGTCTTT CTTCTTTGTGGCGTCCCAGCCTTCAAAAAAG
PTPRU Exon 1 sgRNA Fw	CAGATCATAGTGCGAGGGCT
PTPRU Exon 1 sgRNA Rv	ACACTTGGCATTCACTCGGA
PTPRU Exon 14 sgRNA Fw	TGGGCCCTGTGCTATAGGT
PTPRU Exon 14 sgRNA Rv	AAACAGTCCCAGCAGGCATA
PTPRK-ICD Fw	TGGC[TTCGAA]AAAAAGAGCAAACTTGCTAAAAAACGCAAAGATG
PTPRK-ICD Rv	TGGC[GGTACC]CTAAGATGATTCCAGGTACTCCAAAGCTACATCA
PTPRK.D1057E Fw	CTGGCCTGAACATGGAGTGCC
PTPRK.D1057E Rv	CATGTTCAGGCCAGCCCGTG
PTPRK-D1.ΔpTyr-loop Fw	AATGCCTGCCTATGATCACTCCAGAGTGATTTTGCAACCC
PTPRK-D1.ΔpTyr-loop Rv	CGTCCCAGCCTTCAAAAAAGCTCTCATATTCCTCTTTGAACCCATAG
PTPRK-D1.pTyr loop Fw	AGTATGAGAGCTTCTTTGAAGGACAGTCAGCATCTTGGGATGTAGC
PTPRK-D1.pTyr loop Rv	AGTTTCACTCGGTGCCGATCATATGCTATAATGTTTCCATATCGGTTTTTTGCT CTATTTTG

*Restriction endonuclease sites are marked in parentheses

Supplementary References

1 McParland, V. *et al.* The metastasis-promoting phosphatase PRL-3 shows activity toward phosphoinositides. *Biochemistry* **50**, 7579-7590 (2011).

2 Madeira, F. *et al.* The EMBL-EBI search and sequence analysis tools APIs in 2019. *Nucleic Acids Res* **47**, W636-W641 (2019).
